# Supplementary material for: Myeloid Cell Mobilization and Recruitment by Human Mesothelioma in NSG-SGM3 Mice
Source: Cells. 2024 Dec 23;13(24):2135. doi: 10.3390/cells13242135 (PMC11675005; doi:10.3390/cells13242135)
Supplement: Supplementary file 1 [file cells-13-02135-s001.zip › Supplementary.pdf]

A

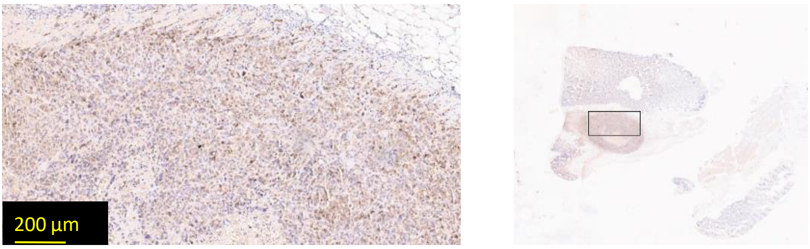

Supplementary Figure S1. Immunohistochemical Wilms Tumor protein (WT1) staining of engrafted mesothelioma. Immunohistochemical staining for WT1 demonstrated strong and diffuse positivity in the majority of tumor cells, supporting the diagnosis of malignant mesothelioma. The images were obtained by Leica Aperio GT450 DX scanner and then processed at 20x magnification using Aperio ImageScope software.

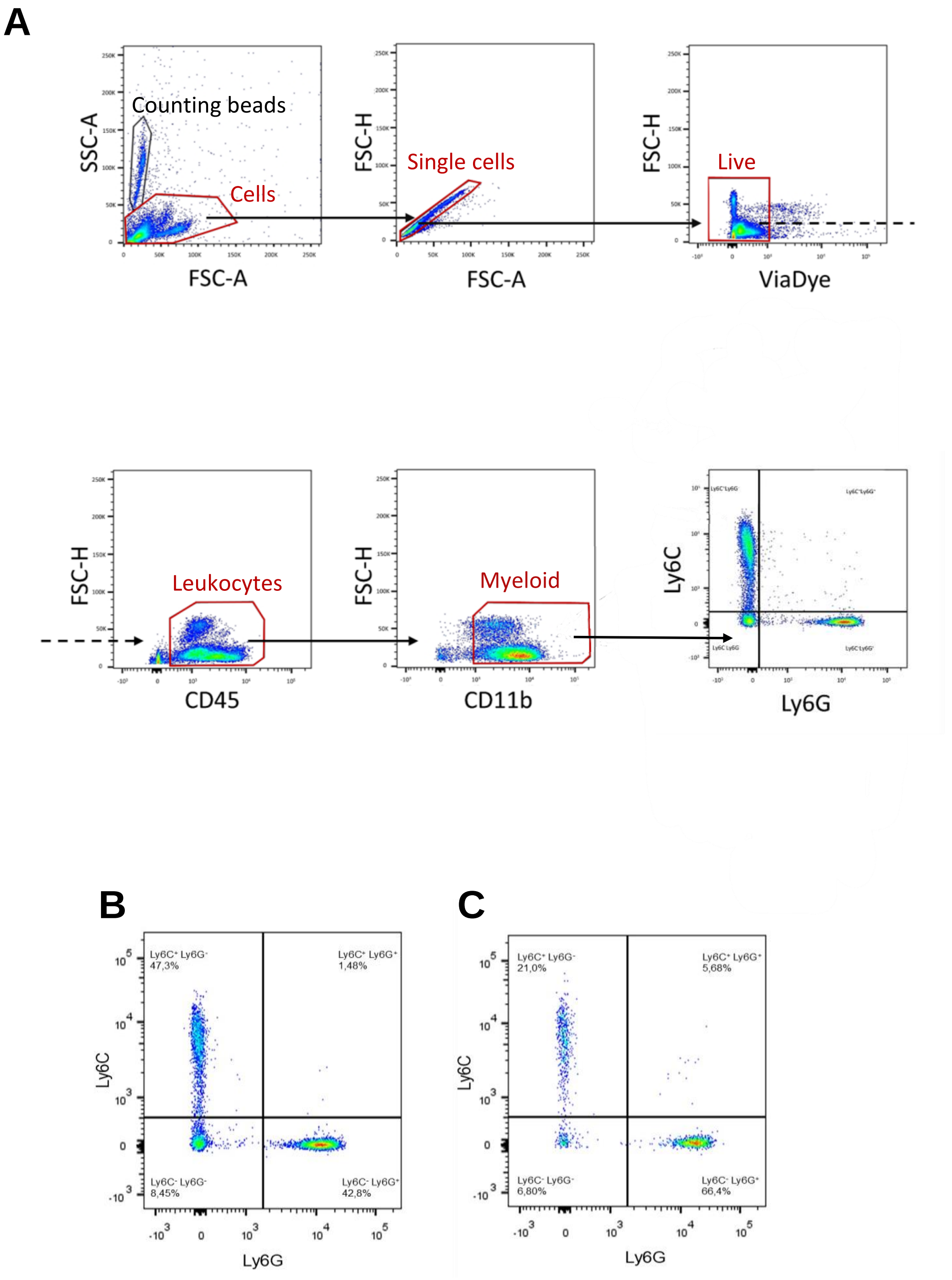

Supplementary Figure S2. Marked increase in the neutrophil-like population in the blood of NSG-SGM3 mice with the engrafted primary mesothelioma. (A) Gating strategy for VD^{-}CD45^{+}CD11b^{+} cells in blood samples of NSG-SGM3 mice. **(B)** Representative flow cytometry dot plots of myeloid populations in naïve and **(C)** in a mesothelioma-bearing NSG-SGM3 mouse. Analysis of myeloid cell populations in naïve NSG-SGM3 mice and the same mice 4 weeks after MZT-Luc2-mCherry cell line implantation.

Characteristics	Cytokine	MZT-Luc2-mCherry	GBM	AML
**Neutrophil differentiation and recruitment**	**G-CSF**	2766,5 ± 159,1	n/d	n/d
	**GM-CSF**	383,7 ± 9,4	4,9 ± 0,2	0,9 ± 0
	**IL-8**	10143,6 ± 54,8	4707,0 ± 122,4	361,5 ± 18,1
	**CXCL1**	15609,6 ± 119,4	3984,0 ± 251,4	28,9 ± 2,2
	**IL-1α**	1094,8 ± 47,5	n/d	8,9 ± 0,8
**Monocyte/macrophage mobilization and recruitment**	**CCL2**	30,3 ± 0,3	10099,6 ± 266,1	8964,2 ± 189,5
	**CCL3**	21,5 ± 0,7	164,0 ± 1,7	17,1 ± 1,6
	**CCL4**	n/d	76,7 ± 6,9	36,2 ± 0,8
	**CCL7**	12,9 ± 0,0	554,8 ± 21,2	8,3 ± 1,5
**Other downregulated cytokines**	**CXCL10**	543,1 ± 3,1	129,2 ± 5,6	2369,6 ± 181,7
	**VEGF**	24,9 ± 4,7	1286,0 ± 232,5	2641,4 ± 126,0
**Other upregulated cytokines**	**FGF-2**	106,3 ± 2,5	56,9 ± 0,0	44,5 ± 4,9
	**Fractalkine**	192,8 ± 16,8	204,6 ± 16,8	77,0 ± 19,8
	**IL-4**	271,6 ± 15,0	175,8 ± 11,4	28,3 ± 9,2
	**Eotoxin**	27,0 ± 0,5	23,3 ± 1,3	9,3 ± 0,8
	**FLT3L**	10,4 ± 0,9	12,2 ± 0,8	6,0 ± 1,2
	**MDC**	27,8 ± 2,9	41,4 ± 5,1	18,9 ± 0,0
	**sCD40L**	21,3 ± 2,4	24,6 ± 2,3	14,1 ± 5,4
	**IFNα2**	17,5 ± 1,7	21,1 ± 1,6	12,2 ± 0,0
	**EGF**	11,2 ± 1,2	10,2 ± 1,0	7,6 ± 0,6
	**IL-7**	15,6 ± 6,1	21,9 ± 0,2	3,3 ± 0,8
**Proinflammatory cytokines**	**IL-1β**	11,5 ± 0,3	3,9 ± 0,1	4,7 ± 0,5
	**IL-1Ra**	n/d	n/d	44,6 ± 9,9
	**IL-6**	74,4 ± 1,6	2594,7 ± 55,0	1,4 ± 0,2
	**TNF**	1,6 ± 0,1	8,5 ± 0,7	70 ± 4,5
**Cytokines with low expression profiles (5 pg/ml and below)**	**TGF-α**	1,1 ± 0,2	2,7 ± 0,1	5 ± 0,1
	**IL-10**	4 ± 0,1	1,3 ± 0,1	0,7 ± 0,5
	**IFN-γ**	0,2 ± 0,1	0,8 ± 0,1	0,5 ± 0,1
	**IL-2**	0,5 ± 0,1	0,9 ± 0,1	0,8 ± 0,0
	**IL-3**	0,1 ± 0,0	0,2 ± 0,1	0,1 ± 0,0
	**IL-5**	n/d	0,6 ± 0,1	0,5 ± 0,0
	**IL-13**	0,8 ± 0,1	1,6 ± 0,0	0,9 ± 0,1
	**IL-12p70**	1,1 ± 0,2	2,0 ± 0,2	1,1 ± 0,0
	**IL-15**	1,8 ± 0,3	2,5 ± 0,2	1,8 ± 0,0
	**Il-12p40**	2,2 ± 0,4	4,4 ± 0,8	4,1 ± 0,7
	**IL-9**	0,9 ± 0,3	3,1 ± 0,3	4,2 ± 0,2
	**Il-17a**	0,6 ± 0,2	2,1 ± 0,0	2,0 ± 0,2

Supplementary Table S1. Cytokine profiles of mesothelioma, glioblastoma, and acute myeloid leukemia cell lines. Cytokine levels (pg/mL) are presented as mean ± SD for each cancer cell line. Measurements were performed using a multiplex assay on cell culture supernatants collected 24 hours after culturing 1 x 10^5 cells in 100 µL of medium. Values below the detection threshold were marked as not detected (n/d). Analytes not present in the assay panel were denoted as not available (n/a).

Characteristics	Cytokine	Murine cytokines		Human cytokines	
		naïve	MZT-Luc2-mCherry	naïve	MZT-Luc2-mCherry
**Neutrophil differentiation and recruitment**	**G-CSF**	23,7 ± 8,0	458,7 ± 1045,4	n/d	8,3 ± 17,5
	**GM-CSF**	n/d	2,0 ± 2,5	6616,0 ± 494,0	6023,5 ± 922,4
	**IL-8**	n/a	n/a	n/d	50,6 ± 42,9
	**CXCL1**	15,4 ± 1,5	59,0 ± 114,4	14,9 ± 4,0	16,3 ± 4,4
	**LIX**	13070,7 ± 938,9	18705,4 ± 5689,0	n/a	n/a
	**CXCL2**	23,8 ± 3,1	23,6 ± 8,7	n/a	n/a
	**IL-1α**	112,2 ± 3,6	185,5 ± 97,0	n/d	n/d
**Monocyte/macro-phage mobilization and recruitment**	**CCL2**	4,4 ± 2,1	42,2 ± 83,7	4,1 ± 8,2	n/d
	**CCL3**	2,3 ± 1,3	5,9 ± 3,1	n/d	n/d
	**CCL4**	n/d	26,9 ± 54,0	n/d	n/d
**Proinflammatory cytokines**	**IL-6**	0,6 ± 0,4	126,6 ± 358,9	n/d	n/d
	**CXCL9**	15,1 ± 16,1	22,9 ± 18,4	n/a	n/a
	**CXCL10**	36,4 ± 8,3	104,3 ± 67,2	n/d	1,2 ± 3,8
**Other cytokines**	**Eotaxin**	620,5 ± 105,6	2171,7 ± 929,0	1,7 ± 2,1	0,6 ± 1,4
	**IL-4**	0,1 ± 0	0,1 ± 0,1	98,3 ± 234,7	n/d
	**IL-3**	n/d	n/d	133,8 ± 21,2	117,5 ± 29,3
	**IL-9**	7,7 ± 2,2	9,2 ± 7,2	0,6 ± 1,4	n/d
	**MDC**	n/a	n/a	5,8 ± 7,3	5,1 ± 5,6
**Cytokines with low expression profiles (5 pg/ml and below)**	**IFNγ**	0 ± 0,1	0,4 ± 1,0	n/d	n/d
	**IL-12p70**	n/d	0,4 ± 0,5	n/d	n/d
	**IL-17a**	n/d	0,2 ± 0,3	0,1 ± 0,0	0,2 ± 0,1
	**IL-15**	0,3 ± 0,2	1,8 ± 1,7	n/d	0,2 ± 0,6
	**VEGF**	n/d	0,1 ± 0,0	0,2 ± 0,4	n/d
	**IL-2**	0,3 ± 0,1	0,3 ± 0,2	n/d	n/d
	**IL-7**	0,1 ± 0,1	0,4 ± 0,4	1,4 ± 1,4	1,8 ± 1,3
	**IL-10**	n/d	0,5 ± 0,7	0,0 ± 0,1	0,0 ± 0,1
	**CCL5**	0,3 ± 0,1	1,6 ± 0,8	n/a	n/a
	**IL-13**	n/d	n/d	0,9 ± 0,6	0,1 ± 0,1
	**M-CSF**	n/d	0,1 ± 0,3	n/a	n/a
	**IL-1β**	0,2 ± 0,2	1,1 ± 0,4	n/d	n/d
	**TNF**	0,4 ± 0,0	1,8 ± 2,2	0,4 ± 0,4	0,0 ± 0,1

Supplementary Table S2. Blood serum cytokine profiles of naïve and tumor-bearing mice. Cytokine levels (pg/mL) are presented as mean ± SD for 4 groups: naïve mice (human and murine cytokines) and 4 weeks after MZT-Luc2-mCherry cell line engraftment (human and murine cytokines). Measurements were performed using a multiplex assay on blood serum samples. Values below the detection threshold were marked as not detected (n/d). Analytes not present in the assay panel were denoted as not available (n/a).

Characteristics	Cytokine	Murine cytokines	Human cytokines
**Neutrophil differentiation and recruitment**	**G-CSF**	48,7 ± 15,4	88,9 ± 45,1
	**GM-CSF**	0,5 ± 0,7	302,1 ± 150,0
	**IL-8**	n/a	2154,6 ± 599,0
	**CXCL1**	30,0 ± 15,8	2645,9 ± 3034,9
	**CXCL2**	868,8 ± 1783,7	n/a
	**IL-1α**	36,6 ± 12,3	325,9 ± 238,0
**Monocyte/macro-phage mobilization and recruitment**	**CCL2**	7,2 ± 7,4	14,3 ± 14,4
	**CCL3**	5,3 ± 3,2	12,5 ± 4,1
**Proinflammatory cytokines**	**IL-1β**	0,2 ± 0,2	34,4 ± 60,2
	**IL-6**	4,0 ± 1,7	13,0 ± 13,1
	**IL-15**	0,5 ± 0,4	30,7 ± 11,3
	**CXCL9**	27,3 ± 43,4	n/a
	**CXCL10**	2,2 ± 1,7	68,1 ± 36,2
**Other cytokines**	**VEGF**	29,3 ± 72,9	62471,3 ± 72065,0
	**Eotaxin**	1,2 ± 2,2	18,6 ± 7,7
	**IL-4**	n/d	85,6 ± 61,7
	**IL-3**	n/d	12,8 ± 15,6
	**IL-7**	n/d	15,0 ± 6,7
	**IL-9**	37,8 ± 18,5	5,6 ± 2,1
	**IL-13**	n/d	6,5 ± 4,7
	**MDC**	n/a	63,4 ± 131,6
**Cytokines with low expression profiles (5 pg/ml and below)**	**TNF**	0,4 ± 0	4,7 ± 4,4
	**IL-12p40**	n/d	4,1 ± 3,0
	**LIX**	2,8 ± 5,9	n/a
	**CCL4**	0,7 ± 2,1	n/d
	**CCL5**	0,2 ± 0,2	n/a
	**M-CSF**	0,2 ± 0,5	n/a
	**IFNγ**	0,2 ± 0,1	1,9 ± 1,2
	**IL-2**	1,4 ± 0,4	0,4 ± 0,3
	**IL-5**	n/d	1,0 ± 0,7
	**IL-10**	n/d	0,6 ± 0,5
	**IL-12p70**	n/d	1,6 ± 1,4
	**IL-17A**	n/d	1,7 ± 1,5

Supplementary Table S3. Cytokine profiles of tumor lysates. Cytokine levels (pg/mL) are presented as mean ± SD for 2 groups: human and murine cytokines 4 weeks after MZT-Luc2-mCherry cell line engraftment. Measurements were performed using a multiplex assay on tumor lysate samples. Values below the detection threshold were marked as not detected (n/d). Analytes not present in the assay panel were denoted as not available (n/a).
